# Supplementary material for: Effectiveness and cost-effectiveness of integrating the management of depression into routine HIV Care in Uganda (the HIV + D trial): A protocol for a cluster-randomised trial
Source: Int J Ment Health Syst. 2021 May 12;15:45. doi: 10.1186/s13033-021-00469-9 (PMC8114695; doi:10.1186/s13033-021-00469-9)
Supplement: Supplementary file 2 — Additional file 2: Appendix 2. Trial management committee. [file 13033_2021_469_MOESM2_ESM.docx]

**Appendix 2: Trial management committees**

**Trial management committees**

| **Committee** | **Role** | **Members** | **Frequency of meeting** |
| --- | --- | --- | --- |
| Independent Data Monitoring Committee (IDMC) | To receive and review information on the progress and accruing data of this trial (including serious adverse events) and provide advice on the conduct of the trial to the Trial Steering Committee. | 1) Prof Crick Lund, Professor of Global Mental Health and Development, King’s College London (Chair)  2) Dr Marc Henrion, Malawi-Liverpool-WT programme (Statistician)  3) Dr Fred Kigozi, Senior Consultant Psychiatrist, Uganda  4) Dr Joshua Tugumisirize, Senior Psychiatrist, Islamic University of Uganda  5) Mr David Mugisha, Ethics expertise, Uganda Virus Research Institute-International AIDS Vaccine Initiative (UVRI-IAVI) | Six monthly intervals |
| Trial Steering Committee (TSC) | To provide oversight for the trial. It provides advice on all aspects of the trial. | **Independent members**  1) Prof Oye Gureje, Psychiatric Epidemiologist, University of Ibadan & Head of WHO Collaborating Centre for Research and Training in Mental Health, Neurosciences and Substance Abuse, Nigeria (Chairperson)  2) Dr Arthur Ahimbisibwe, National Coordinator Care and Treatment at the AIDS Control Programme, MOH, Uganda  3) Prof David Ndetei, Senior Psychiatric Epidemiologist, Nairobi University & Head of the African Foundation, Kenya  4) Ms. Betty Ndagire, Represents users  **Non-independent Investigator members**  1) Prof Eugene Kinyanda, Psychiatric Epidemiologist & Head of Mental Health Section, MRC/UVRI & LSHTM– PI  2) Prof Vikram Patel, Psychiatric Epidemiologist, Harvard University - Co-Sponsor  3) Prof Melissa Neuman, LSHTM – Trial Statistician  4) Dr Richard Mpango, Clinical Psychologist, Mental Health Section, MRC/UVRI & LSHTM-TMT Facilitator  5) Dr Leticia Kyohangirwe, Psychiatrist, Mental Health Section, MRC/UVRI & LSHTM– TMT Facilitator | Six monthly intervals |
| Trial Management Team (TMT) | Responsible for the day to day running of the trial | PI, Project Leads, other scientists involved in implementing the study (psychologists, statisticians, health economists, data managers) and administrators (including the Project Manager) | Weekly |
| The Trial Management Group (TMG) | The TMG is responsible for the management of the trial | Comprises of members of the TMT, all collaborators, Ministry of Health representatives and community representatives. | Monthly |
